# Validated Wearable Device Shows Acute Postoperative Changes in Sleep Patterns Consistent With Patient-Reported Outcomes and Progressive Decreases in Device Compliance After Shoulder Surgery

**DOI:** 10.1016/j.asmr.2023.100783

**Published:** 2023-08-14

**Authors:** Pranav V. Gadangi, Bradley S. Lambert, Haley Goble, Joshua D. Harris, Patrick C. McCulloch

**Affiliations:** a Texas A&M Health Science Center, College Station, Texas, U.S.A.; b Texas A&M College of Engineering, College Station, Texas, U.S.A.; c Houston Methodist Orthopedics & Sports Medicine, Houston Methodist Hospital, Houston, Texas, U.S.A.

## Abstract

**Purpose:**

To assess the utility of a validated wearable device (VWD) in examining preoperative and postoperative sleep patterns and how these data compare to patient-reported outcomes (PROs) after rotator cuff repair (RCR) or total shoulder arthroplasty (TSA).

**Methods:**

Male and female adult patients undergoing either RCR or TSA were followed up from 34 days preoperatively to 6 weeks postoperatively. Sleep metrics were collected using a VWD in an unsupervised setting. PROs were assessed using the following validated outcome measures: Patient-Reported Outcomes Measurement Information System (PROMIS) Physical Function questionnaire; American Shoulder and Elbow Surgeons self-evaluation questionnaire; visual analog scale assessing pain; and Disabilities of the Arm, Shoulder and Hand questionnaire. Data were analyzed preoperatively and at 2-week intervals postoperatively with χ^2^ analysis to evaluate device compliance. Sleep metrics and PROs were evaluated at each interval relative to preoperative values within each surgery type with an analysis of variance repeated on time point. The relation between sleep metrics and PROs was assessed with correlation analysis.

**Results:**

A total of 57 patients were included, 37 in the RCR group and 20 in the TSA group. The rate of device compliance in the RCR group decreased from 84% at surgery to 46% by 6 weeks postoperatively (*P* < .001). Similarly, the rate of device compliance in the TSA group decreased from 81% to 52% (*P* < .001). Deep sleep decreased in RCR patients at 2 to 4 weeks (decrease by 10.99 ± 3.96 minutes, *P* = .021) and 4 to 6 weeks postoperatively (decrease by 13.37 ± 4.08 minutes, *P* = .008). TSA patients showed decreased deep sleep at 0 to 2 weeks postoperatively (decrease by 12.91 ± 5.62 minutes, *P* = .045) and increased rapid eye movement sleep at 2 to 4 weeks postoperatively (increase by 26.91 ± 10.70 minutes, *P* = .031). Rapid eye movement sleep in the RCR group and total sleep in the TSA group were positively correlated with more favorable PROs (*P* < .05).

**Conclusions:**

VWDs allow for monitoring components of sleep that offer insight into potential targets for improving postoperative fatigue, pain, and overall recovery after shoulder surgery. However, population demographic factors and ease of device use are barriers to optimized patient compliance during data collection.

**Level of Evidence:**

Level IV, diagnostic case series.

Sleep deprivation is known to decrease pain tolerance, which can negatively impact the progression of postoperative rehabilitation.^1^ The resultant postoperative fatigue can negatively impact clinical outcomes by limiting a patient’s ability to perform activities of daily living and overall return to activity.^2^^,^^3^ Assessing such factors in shoulder patients is essential because sleep disturbance is one of the most common symptoms observed preoperatively and postoperatively.^4^^,^^5^

Up to this point, assessments of the effects of sleep deprivation experienced by outpatient shoulder surgery patients in the preoperative and postoperative periods on postoperative pain and fatigue have been primarily established with subjective, validated outcome measure surveys.^4^^,^^5^ Austin et al.^4^ showed that prior to surgery, 89% of patients undergoing rotator cuff repair (RCR) reported Pittsburgh Sleep Quality Index scores indicative of sleep deprivation, which did not show significant improvement until 3 months after the procedure. In patients undergoing total shoulder arthroplasty (TSA), Morris et al.^5^ observed that the prevalence of sleep disturbance decreased from 91% preoperatively to 20% postoperatively, and the decrease was associated with improved patient-reported outcomes (PROs).

Despite the established relation between postoperative sleep quality and patient recovery, no study has objectively measured sleep to assess this relation. Considering that rapid eye movement (REM) sleep functions to restore spatial and procedural memory after learning new motor skills and that deep sleep augments the body’s restoration, objective data describing the time spent in these stages are integral to interpreting the effects of sleep deprivation on postoperative recovery.^6^, ^7^, ^8^ As the number of orthopaedic procedures considered “outpatient” procedures has been increasing, validated wearable devices (VWDs) have gained considerable interest for noninvasive, at-home monitoring of these sleep metrics and other physiological parameters in postoperative populations. VWDs vary in their proprietary algorithms but are often similar in their use of heart rate measures, including resting heart rate and heart rate variability, to assess sleep. The fluctuations and patterns of these values throughout the night help determine the stage of sleep.^9^, ^10^, ^11^, ^12^

Although RCR and TSA are different procedures, they have some similarities. For instance, the pain associated with rotator cuff tears and shoulder arthritis is the primary cause of sleep disturbance, which occurs in similar age groups, and both procedures are now becoming managed as outpatient surgical procedures.^13^, ^14^, ^15^ The purpose of this study was to assess the utility of a VWD in examining preoperative and postoperative sleep patterns and how these data compare to PROs after RCR and TSA. We hypothesized that the study would reveal the following: Patient compliance with the device would be high. Acute changes in sleep stage data and sleep quantity from preoperative baselines would be seen in the early postoperative period, with improvement by postoperative week 6. There would be a significant correlation between sleep metrics and more favorable PROs.

## Methods

This study received institutional review board approval prior to initiation. Patients who underwent either RCR for rotator cuff arthropathy or TSA (anatomic or reverse) for glenohumeral arthritis between February 2018 and May 2019 were retrospectively identified. The inclusion criteria required enrolled patients to be aged 18 years or older. Rotator cuff arthropathy was due to both acute traumatic tears and degenerative tears. Patients in both groups complained of substantial sleep disturbance, such as having difficulty falling and staying asleep, due to shoulder pain prior to surgery. A single, experienced attending orthopaedic surgeon (P.C.M.) conducted all procedures between February 2018 and May 2019. The anesthesia protocol for all procedures included general anesthesia and an interscalene block. All patients underwent same-day discharge (i.e., discharge within 24 hours). Postoperative pain management in all patients consisted of 40 narcotic pills without refills. The surgeon instructed all patients to continue sling use for 6 weeks when not performing rehabilitative exercises. All patients were prescribed supervised outpatient physical therapy that started 2 weeks after surgery. Patients undergoing revision surgery or planned inpatient stays (owing to medical comorbidities) met our exclusion criteria and were excluded from the study.

### Patient-Reported Outcomes

Preoperative and postoperative fatigue and pain were assessed with 4 validated outcome surveys: Patient-Reported Outcomes Measurement Information System (PROMIS) Physical Function questionnaire^16^; American Shoulder and Elbow Surgeons (ASES) self-evaluation questionnaire^17^^,^^18^; Disabilities of the Arm, Shoulder and Hand (DASH) questionnaire^17^^,^^18^; and visual analog scale (VAS) assessing pain.^19^ We instructed participants to complete these questionnaires and turn them in preoperatively on the day of surgery and postoperatively at weeks 2, 4, and 6.

### Wearable Device Sleep Monitoring

Sleep metrics were measured using a WHOOP 2.0 Strap, validated to measure sleep duration, assess 4-stage sleep against high-level polysomnography, and assess 2-stage sleep against high-level actigraphy.^9^^,^^10^^,^^20^ The WHOOP device uses a 3-axis accelerometer and reflectance photoplethysmography to measure 5 metrics—heart rate, heart rate variability, ambient temperature, motion and movement, and skin response—to measure and analyze a person’s sleep. The partnered mobile application reports sleep data as total time spent in bed and time spent awake, in light sleep, in REM sleep, and in deep sleep. Participants were blinded to these data throughout the study.

**Fig 1:**
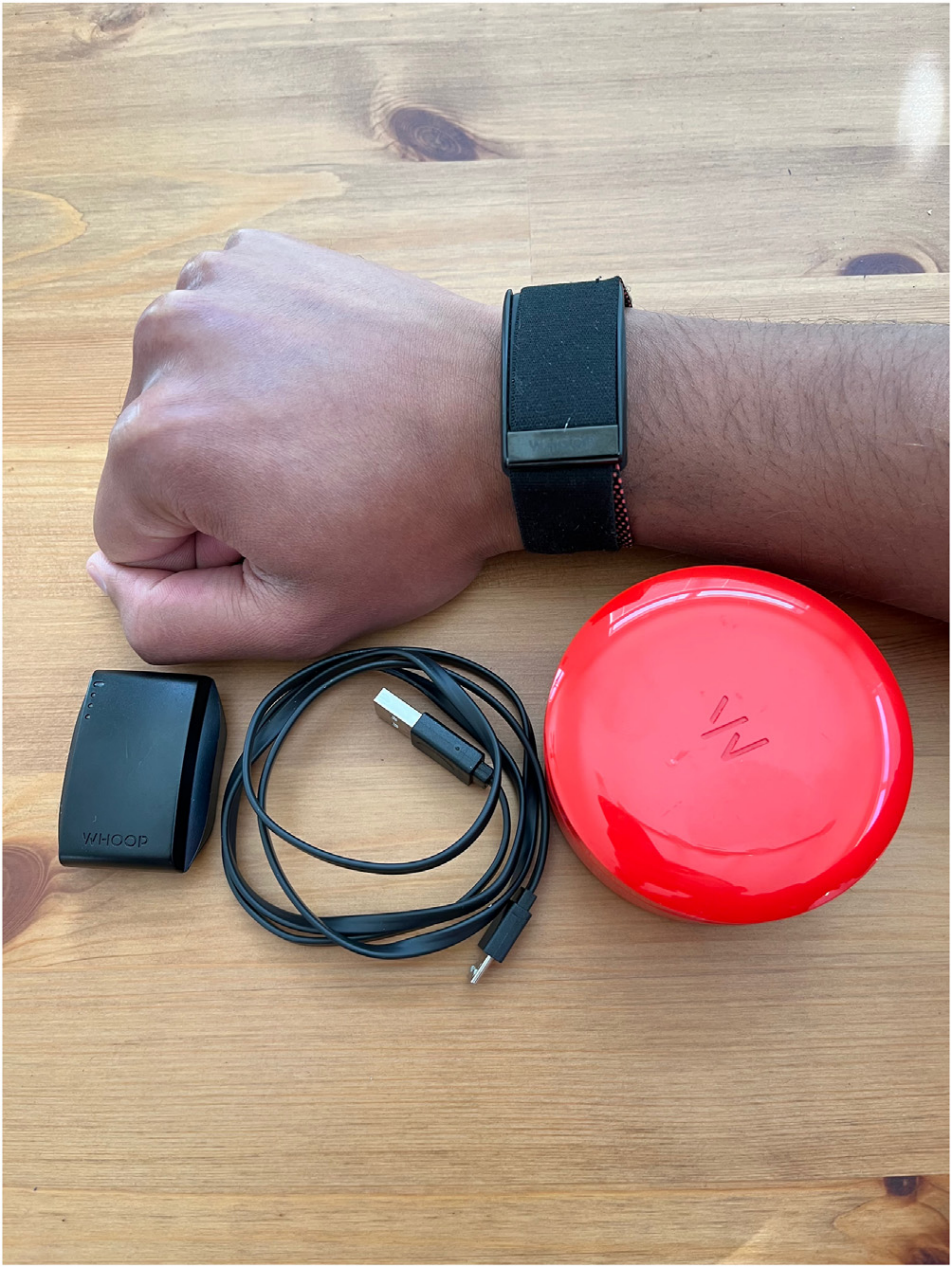
WHOOP device and accessories: battery charging pack, charging cable, and case.

At study initiation, participants were provided 1 WHOOP 2.0 Strap, 1 WHOOP battery charging pack, 1 WHOOP charging cable, an instructional handout, and an instructional video created by us (Fig 1). The WHOOP device was selected because it is a wearable technology that does not have a patient-facing screen that could bias patients’ self-reporting. It also has a “team” dashboard function through which researchers can track all participants. The handout and session covered basic use, working with the partnered mobile application, and troubleshooting. Participants were instructed to contact us immediately if any difficulties arose with using the WHOOP device or its accessories during the study period. The participants were followed up from 34 days before surgery to 6 weeks after the procedure. This time was divided into 2 phases: preoperative phase (from 34 days preoperatively until surgery) and postoperative phase (from the day of surgery until 6 weeks postoperatively). Participants were instructed to wear the WHOOP device at all times during the study period and to sync the device daily with the partnered mobile application. Daily WHOOP sleep metrics were then recorded. All data collection occurred in each participant’s home. After the 6-week study period, all WHOOP devices were collected for data analysis of sleep metrics and device compliance, which required sleep metrics to be correctly recorded for each postoperative week. To be considered compliant for data collection, participants were required to successfully wear the device for a minimum of 3 full days (72 hours) per week. Sleep metrics were averaged across the entire preoperative period and then across 2-week intervals after surgery.

### Statistical Analysis

Statistical analyses were performed using SPSS Statistics software (version 23.0; IBM). Sleep metrics were analyzed for the preoperative period and in 2-week postoperative intervals (0-2 weeks, 2-4 weeks, and 4-6 weeks). First, χ^2^ analysis was used to analyze WHOOP device compliance for all patients within each surgery type. Next, for all patients who remained compliant throughout the study, sleep metrics (measured in absolute time and percentage of total sleep time) were analyzed using an analysis of variance repeated on time (4 time points) within each surgery type. Similarly, the Friedman test for nonparametric data followed by the Wilcoxon signed rank test was used to compare clinical outcome scores (VAS, DASH, ASES, and PROMIS Physical Function) within each surgery type preoperatively and at the 2-, 4-, and 6-week postoperative time points. For sleep metrics and PROs, significant effects indicated by type III tests of fixed effects were followed by a Bonferroni post hoc test for pair-wise comparisons within surgery types compared with preoperative measures. For all significant pair-wise comparisons, the effect size (ES) was calculated using the Cohen *d* statistic, whereby type I error was set at α = .05 for all analyses. After post hoc analyses, for all significant pair-wise comparisons, the ES was calculated using the Cohen *d* statistic,^21^ whereby ES data were interpreted as follows: 0.0 to 0.1, negligible; 0.1 to 0.3, small; 0.3 to 0.5, moderate; 0.5 to 0.7, large; and greater than 0.7, very large.^22^, ^23^, ^24^ Finally, Spearman rank correlation analysis was used to determine whether sleep metrics correlated with PROs for all matched–time point measures during the study period.

## Results

### Device Compliance

**Fig 2:**
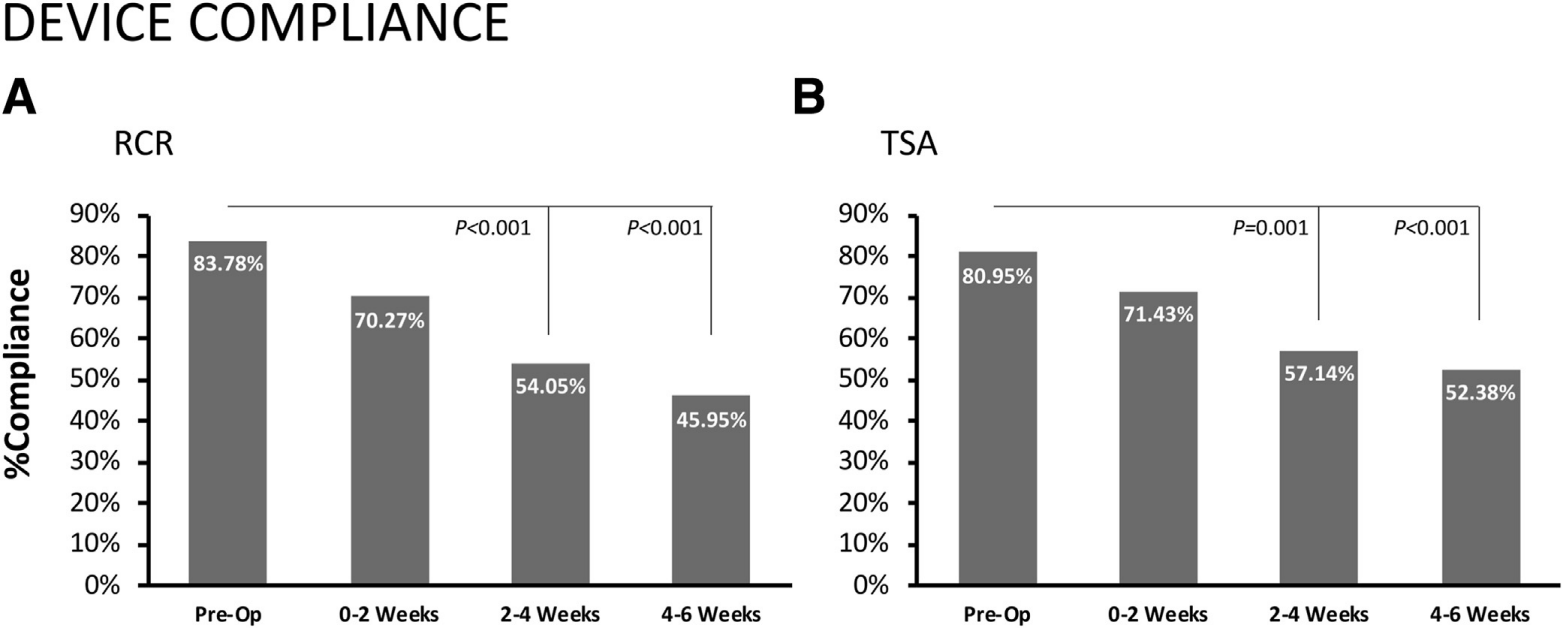
Device compliance. Data are presented as frequencies for patients undergoing rotator cuff repair (RCR, A) or total shoulder arthroplasty (TSA, B) who remained compliant with wearing WHOOP device during each measurement period. (Pre-Op, preoperatively.)

A total of 57 patients participated in this investigation, with 37 in the RCR group and 20 in the TSA group (Table 1). Data on device compliance within each surgery type are shown in Figure 2. Within both surgery types, the rate of compliance decreased compared with the preoperative measurement period by the 2- to 4-week and 4- to 6-week intervals (Fig 2, *P* < .05). The patient-reported causes of noncompliance included nonwear, poor connectivity between the WHOOP device and partnered mobile application, and irregular charging of the WHOOP device.

**Table 1:** Age Data by Group and Overall

	RCR		TSA		Total (N = 57)
	Men (n = 20)	Women (n = 17)	Men (n = 11)	Women (n = 9)	
Age, mean ± SD (range), yr	60.85 ± 7.94 (47-74)	57.41 ± 9.19 (42-70)	68.18 ± 5.71 (60-78)	62.67 ± 10.12 (44-80)	61.53 ± 8.89 (42-80)

NOTE. Patients underwent RCR for the treatment of acute traumatic or degenerative rotator cuff tears. Patients underwent TSA (anatomic or reverse) for the treatment of glenohumeral arthritis.

RCR, rotator cuff repair; SD, standard deviation; TSA, total shoulder arthroplasty.

### Sleep Metrics

WHOOP device–reported sleep metrics for all patients remaining compliant during the study period (45.95% in RCR group and 52.38% in TSA group) are presented in Table 2. Among RCR patients, deep sleep was observed to be decreased at the 2- to 4-week interval (*P* = .021, decrease by 10.99 ± 3.96 minutes, ES = 0.33 [moderate]) and 4- to 6-week interval (*P* = .008, decrease by 13.37 ± 4.08 minutes, ES = 0.40 [moderate]) relative to preoperatively. Among TSA patients, deep sleep was observed to decrease compared with preoperatively at the 0- to 2-week postoperative interval (*P* = .045, decrease by 12.91 ± 5.62 minutes, ES = 0.40 [moderate]). However, this was followed by an increase in total sleep time relative to preoperatively at the 2- to 4-week interval (*P* = .007, increase by 30.63 ± 8.96 minutes, ES = 0.39 [moderate]) and 4- to 6-week interval (*P* = .046, increase by 24.79 ± 11.50 minutes, ES = 0.29 [small]). TSA patients also experienced an increase in REM sleep at the 2- to 4-week interval (*P* = .031, increase by 26.91 ± 10.70 minutes, ES = 0.30 [moderate]).

**Table 2:** Sleep Metrics

Sleep Metric	Preoperatively	Postoperatively		
		0-2 wk	2-4 wk	4-6 wk
Total sleep, h				
RCR	6.03 ± 0.67	5.78 ± 0.81	6.05 ± 0.52	5.81 ± 0.44
TSA	5.66 ± 0.82	5.93 ± 0.63	6.17 ± 0.73∗∗	6.07 ± 0.84∗
REM sleep, h				
RCR	1.43 ± 0.45	1.48 ± 0.45	1.62 ± 0.46	1.52 ± 0.45
TSA	1.55 ± 0.78	1.79 ± 0.45	2.00 ± 0.97∗	1.81 ± 0.98
Deep sleep, h				
RCR	1.15 ± 0.33	0.96 ± 0.34	0.94 ± 0.28∗	0.90 ± 0.26∗∗
TSA	0.73 ± 0.35	0.51 ± 0.30∗	0.57 ± 0.30	0.74 ± 0.35
Light sleep, h				
RCR	3.45 ± 0.38	3.34 ± 0.52	3.48 ± 0.45	3.39 ± 0.40
TSA	3.38 ± 0.80	3.63 ± 0.84	3.60 ± 0.90	3.51 ± 0.82

NOTE. Data are presented as mean ± 95% confidence interval for sleep phase duration (in hours) for all patients who met minimum compliance for the entire postoperative period.

RCR, rotator cuff repair; REM, rapid eye movement; TSA, total shoulder arthroplasty.

∗ Significantly different from preoperatively within sleep phase type at *P* < .05.

∗∗ Significantly different from preoperatively within sleep phase type at *P* < .01.

### Patient-Reported Clinical Outcomes

**Fig 3:**
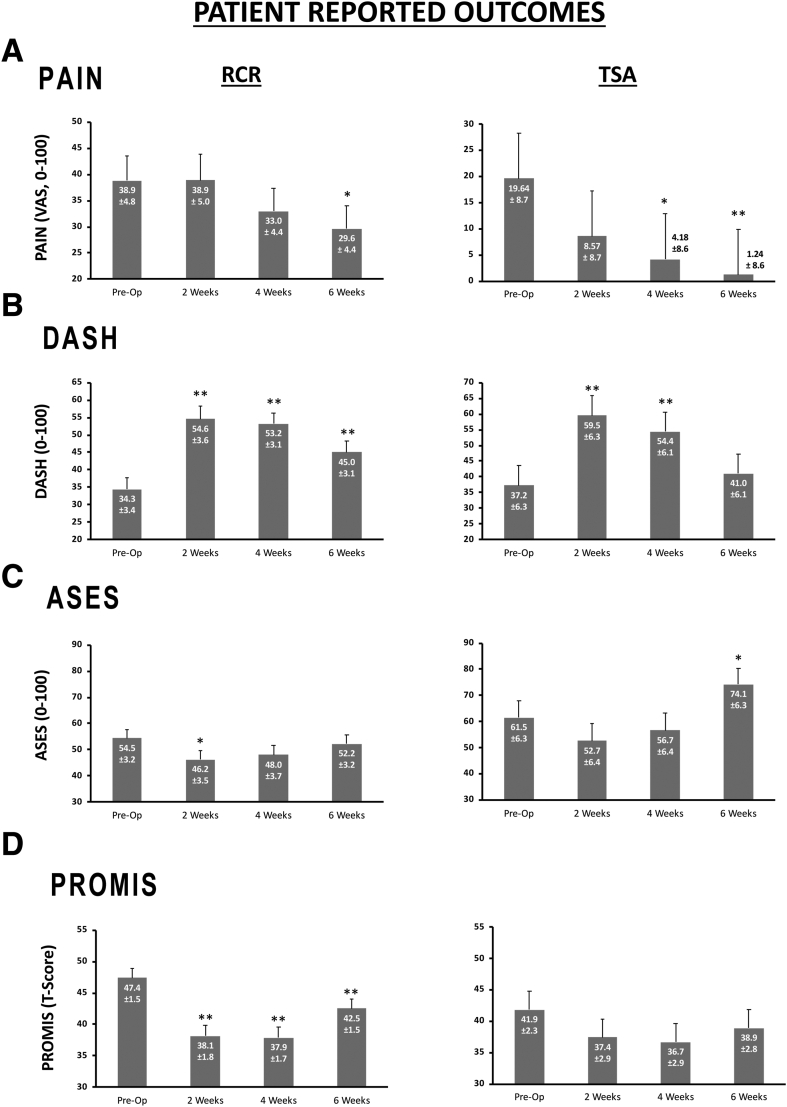
Patient-reported outcome scores. Data are presented as mean ± standard error of the mean for patient-reported pain (visual analog scale [VAS] score, A) as well as survey responses to the Disabilities of the Arm, Shoulder and Hand (DASH) questionnaire (B), American Shoulder and Elbow Surgeons (ASES) self-evaluation questionnaire (C), and Patient-Reported Outcomes Measurement Information System (PROMIS) Physical Function questionnaire (D). Asterisks indicate significant differences from preoperatively (Pre-Op) within sleep phase type and surgery type at *P* < .05 (1 asterisk) and *P* < .01 (2 asterisks).

PRO measures are presented in Figure 3. A significant within–surgery type pain reduction was observed at week 4 for TSA (*P* = .021, ES = 0.83 [very large]), at week 6 for TSA (*P* = .006, ES = 1.29 [very large]), and at week 6 for RCR (*P* = .034, ES = 0.38 [moderate]) relative to preoperative pain levels (Fig 3A). Both surgery types were observed to have an increased DASH score relative to baseline at 2 and 4 weeks postoperatively (*P* < .05, ES > 0.5 [large]) (Fig 3B). In addition, DASH scores remained above preoperative values at 6 weeks after RCR (*P* = .001, ES = 0.59 [large]). ASES scores for TSA patients were increased at postoperative week 6 compared with the preoperative assessment (*P* = .009, ES = 0.84 [very large]) (Fig 3C). Additionally, RCR patients experienced a decrease in ASES scores at postoperative week 2 relative to preoperative values (*P* = .019, ES = 0.43 [moderate]) that returned to baseline at postoperative weeks 4 and 6 (Fig 3C). No detectable changes in PROMIS Physical Function scores were observed after TSA. After RCR, PROMIS Physical Function scores were observed to decrease relative to preoperative measures at all postoperative measurement time points (*P* < .01, ES > 0.5 [large]) (Fig 3D).

### Relation Between Sleep Phases and PROs

The results of our correlation analysis between all matched–time point sleep and outcome measures are shown in Table 3. Outcome scores were significantly—but differentially—correlated with sleep metrics recorded for RCR and TSA patients. RCR PROs for the DASH score (negative correlation), ASES score (positive correlation), and PROMIS Physical Function score (positive correlation) were significantly correlated with REM sleep time, indicating that greater total REM sleep was correlated with more favorable PROs (*P* < .05). In contrast, TSA PROs were observed to be significantly correlated with total sleep time for the pain, DASH, and PROMIS Physical Function scores, with greater total sleep time being correlated with more favorable PROs (*P* < .05). No significant correlations were observed between sleep phase and light sleep, deep sleep, or percentage of each sleep phase relative to total sleep time.

**Table 3:** Correlation Between Sleep Metrics and Patient-Reported Outcomes

	RCR		TSA	
	Total Sleep Time	REM Sleep Time	Total Sleep Time	REM Sleep Time
Pain score				
r Value	–0.128	–0.120	–0.383∗∗	–0.191
P value	.304	.335	.009	.209
DASH score				
r Value	–0.126	–0.378∗∗	–0.401∗∗	–0.162
P value	.313	.002	.006	.288
ASES score				
r Value	0.018	0.253∗	0.282	0.209
P value	.889	.044	.06	.169
PROMIS Physical Function score				
r Value	0.198	0.362∗∗	0.346∗	0.099
P value	.117	.003	.02	.517

PROMIS, Patient-Reported Outcomes Measurement Information System; RCR, rotator cuff repair; REM, rapid eye movement; TSA, total shoulder arthroplasty.

∗ Correlation at *P* < .05.

∗∗ Correlation at *P* < .01.

## Discussion

The most important finding from this study was that compliance with the WHOOP VWD significantly decreased throughout the study and must be considered when using a VWD to monitor sleep. It is possible that as patients experienced a subjective improvement in sleep or pain, they became less compliant with wearing the device, causing a reporting bias. In line with our hypotheses, the VWD was able to show acute changes in patients’ preoperative deep and REM sleep in the early postoperative period. Acute changes included decreased deep sleep in the first 2 postoperative weeks and increased REM sleep by 2 to 4 weeks postoperatively. The collected data showed no change in total sleep during the initial postoperative period. The observed sleep architecture showed no significant difference between the RCR and TSA groups, likely owing to identical anesthesia and postoperative pain management protocols. On the other hand, correlation analysis between sleep metrics and favorable PROs showed that the 2 procedure types varied in which sleep metrics exhibited the observed positive correlations. These results support using a VWD in an outpatient setting to collect sleep data to determine which components of the sleep cycle may be most associated with PROs.

### Sleep Stages

Sleep is generally split into REM and non-REM sleep. Non-REM sleep is further divided into 3 stages labeled N1, N2, and N3, with deep sleep defined as stages N2 and N3.^25^ Deep sleep generally makes up roughly 60% of total sleep, whereas REM sleep makes up roughly 20%. A healthy adult would be expected to have 4 to 6 full non-REM–REM cycles in a single night, with REM time peaking during the last third of the night.^26^ In normal aging adults, a decrease in total sleep time by roughly 10 minutes per decade is expected after 30 years of age.^27^ In addition, Keklund and Akerstedt^28^ showed positive correlations between sleep quality index scores and both deep sleep and REM sleep. Therefore, the assessment of changes of this magnitude within individual stages during a 6-week period was determined to be an effective measure of sleep quality.

### Patient Compliance

Studies conducted using VWDs have often experienced a degree of decreased compliance with their respective devices, even in controlled settings.^10^^,^^29^, ^30^, ^31^, ^32^ Therefore, compliance with the WHOOP device was a concern. The rate of compliance was 100% on day 1 of the preoperative period and decreased to 81% by the surgery date. Although this led us to expect an insignificant amount of decreased compliance over time, we observed significant decreases, particularly at the 2- to 4-week and 4- to 6-week time points (Fig 2). Efforts to mitigate the decreased compliance with the WHOOP device included phone calls and e-mail conversations with participants who had difficulties. Cho et al.^32^ explored challenges experienced when using VWDs for data collection and described 3 categories into which the challenges could fall: device- and technical-related factors, user-related factors, and data governance–related factors. Although considerations of these categories were not implemented into the protocol when our study was conducted, a brief note of each difficulty brought to our attention was taken. From informal assessment, we observed that most of the issues experienced fell into the categories of “device- and technical-related factors” and “user-related factors,” with “poor connectivity” and “non-wear” being the most common issues, respectively. The issues were likely experienced because of the relatively older age of the population studied (mean age, 61.53 years), limited battery life (36 hours), and lack of an interactive screen on the device (Table 1). Further assessment is required to confirm these potential causes of decreased patient compliance with the WHOOP device, which investigators could likely target by providing daily reminders regarding charging, wear, and syncing with the mobile application, as well as an extensive troubleshooting guide. The WHOOP device can be used to assess sleep in an outpatient orthopaedic setting; however, population demographic factors and ease of device use play a significant role in the completeness and quality of data collected.

### Acute Sleep Changes

Decreases in total nightly sleep with normal aging are well established. According to Li et al.,^33^ healthy adults older than 60 years, on average, experience 8.1 hours of total nightly sleep relative to their middle-aged counterparts, who experience 9.1 hours. Although elderly patients experience this decreased sleep, they still exceed the acceptable minimum threshold of 7 hours set by the American Academy of Sleep Medicine and Sleep Research Society.^34^ Achieving fewer than 7 hours of nightly sleep can increase pain and impair daily performance.^34^ Our study population achieved around 27% less sleep than healthy adults older than 60 years and around 16% less sleep than recommended during the preoperative period (Table 2). Additionally, this population spent 6% more time in REM sleep and 6% less time in deep sleep from the preoperative period (Table 2). These findings provide objective evidence that shoulder pain can lead to sleep deprivation preoperatively, and specifically, these data identify which stages are primarily affected. These findings also suggest that the patients in our study may have developed a new baseline prior to surgery. Within the first 6 weeks of the postoperative period, patients did not reach population-level standards. This is an essential educational point for patients undergoing RCR or TSA because it can temper expectations regarding when patients can begin to see sleep improvement. Longer-term studies may be helpful in determining when patients will return to preoperative baselines and population standards for healthy adults older than 60 years.

Sleep deprivation and postoperative fatigue have been noted after various major surgical procedures.^35^, ^36^, ^37^, ^38^ Caravan et al.^39^ concluded that pain could be diagnosed and targeted by assessing the density of sleep spindles. Because sleep spindles are most prominent during deep sleep, it can be inferred that a decrease in deep sleep could indicate a lower pain threshold. This observation is consistent with the findings of studies presented in a theoretical review of postoperative sleep conducted by Chouchou et al.,^40^ as well as a study performed by Onen et al.^41^ Although our study did not show a significant decrease in total sleep time postoperatively, it showed significant, acute worsening of DASH and ASES scores and a significant decrease in deep sleep in the 0- to 2-week postoperative period (Fig 3). These data imply that close monitoring of time explicitly spent in deep sleep could indicate which patients are likely to require more intensive postoperative pain management in this population.

Buchegger et al.^6^ established in trampolining that procedural learning sessions, similar to what is performed during physical therapy, are followed by enhanced REM sleep. An increase in REM sleep was consistent with the initiation of supervised outpatient physical therapy in these patients, which occurred based on the recommendations of the American Society of Shoulder and Elbow Therapists.^42^^,^^43^ Therefore, monitoring changes in postoperative REM sleep may offer insight into how RCR and TSA patients progress through rehabilitation programs; however, further assessment is necessary to stratify this association from confounding factors such as REM sleep improvement due to time from surgery.

### Sleep and PROs

Previous studies have shown favorable correlations between improved subjective sleep quality and postoperative pain and function.^4^^,^^44^, ^45^, ^46^ On the basis of the data collected in this study, we observed similar favorable correlations between objective sleep data and 3 of the 4 validated outcome surveys in both groups; however, the correlations differed based on procedure type (correlation with REM sleep in RCR group vs correlation with total sleep in TSA group). Additionally, the 2 groups differed in that only the TSA group showed a correlation with VAS scores and only the RCR group showed a correlation with ASES scores (Table 3). RCR was performed arthroscopically in the population studied, whereas TSA was performed as an open procedure. The different approaches may have accounted for some of the differences seen. Another possible explanation could be interindividual differences between groups when completing the outcome measure surveys. Although this study cannot determine the exact cause of this difference, future studies could assess the effect of procedure type on sleep architecture and quality.

### Limitations

This study was not without limitations. As discussed previously, a major limitation of this study was poor patient compliance with the WHOOP device, likely owing to unfamiliarity with the technology by the study population and lack of a display screen on the device. Although an advantage of this device is that patients cannot directly observe their sleep data (as not to influence the results), poor device compliance limits its clinical efficacy. Therefore, clinicians and researchers should consider these limitations when choosing the appropriate sleep-tracking device. Similarly, this study did not incorporate a formal method for assessing participants’ reasoning for poor compliance. For future studies hoping to use VWDs in an outpatient orthopaedic setting, a method should be in place to guide improvements in experimental design. Our study is also limited in that it does not consider the central nervous system effects of postoperative multimodal analgesic regimens, which have been shown to influence sleep quality.^47^ Additionally, it does not include the effects of each patient’s home environment on his or her postoperative sleep quality or the patients’ perceptions of their sleep quality. Further studies are required to assess which factors play the most considerable role in postoperative sleep deprivation to optimize patient recovery for outpatient orthopaedic procedures and to determine how subjective assessments of sleep may relate to quantitative objective measures obtained from VWDs in this patient population. Finally, many of the questions among the patient outcome surveys are “functional” in nature. Because patients are often wearing a sling in the first few weeks after surgery, the utility of the functional questions may be highly limited and composite scores during the study period were expectedly low. However, given that the surveys also have questions regarding pain management, pain medication, general daily living tasks, quality of life, and mental status, we believed that determining how sleep metrics correlate to the survey scores would be clinically meaningful particularly during the period when sleep disturbances tend to be most common after shoulder surgery.

## Conclusions

VWDs allow for monitoring components of sleep that offer insight into potential targets for improving postoperative fatigue, pain, and overall recovery after shoulder surgery. However, population demographic factors and ease of device use are barriers to optimized patient compliance during data collection.

## Supplementary Data

ICMJE author disclosure forms
